# Medication-Related Osteonecrosis of the Jaw: A Systematic Review of Case Reports and Case Series

**DOI:** 10.3390/diseases12090205

**Published:** 2024-09-09

**Authors:** Filipa Frutuoso, Filipe Freitas, Miguel Vilares, Helena Francisco, Duarte Marques, João Caramês, André Moreira

**Affiliations:** 1 Faculdade de Medicina Dentária, Universidade de Lisboa, 1600-277 Lisbon, Portugal; 2 Department of Oral Surgery and Oral Medicine, Faculdade de Medicina Dentária, Universidade de Lisboa, 1600-277 Lisbon, Portugal; 3 Department of Head and Neck Surgery, Instituto Português de Oncologia de Lisboa, 1099-023 Lisbon, Portugal; 4 Oral Biology and Biochemistry Research Group, Faculdade de Medicina Dentária, Universidade de Lisboa, 1600-277 Lisbon, Portugal

**Keywords:** medication, MRONJ, osteonecrosis, jaw

## Abstract

Medication-related osteonecrosis of the jaw (MRONJ) is a rare condition, typically seen in patients receiving antiresorptive or antiangiogenic drugs. This study aims to synthesize reports and case series of MRONJ regarding sociodemographic and clinical characteristics and to evaluate the effectiveness of the treatments applied. Following PRISMA guidelines, a search for case reports and case series was carried out in the PubMed-Medline database until March 2024. A total of 88 articles were included in this review, in a total of 151 cases. The key findings reveal that females were the most affected individuals (71% of the cases) with the average age at diagnosis being 66.27 years with a standard deviation of ±13.03. Stage 2 was the most observed stage, in 43% of cases, and zoledronic acid was the most commonly used drug (32% of cases). The oral route was the most common route of administration, in 26% of cases, with an average administration duration of 60.88 months (standard deviation ± 50.92). The mandible was the most commonly affected anatomical location (in 60% of cases). Amoxicillin + clavulanic acid (875 mg + 125 mg) and chlorhexidine (0.12%) were the most used antibiotics and mouthwash, with 16% and 26% of the cases, respectively. Surgical debridement was the most common surgical procedure, in 32% of cases, while the use of an L-PRF membrane was the most prevalent unconventional treatment, in 21% of cases. This study identified a statistically significant relationship between unconventional treatments and the cure of MRONJ (*p* < 0.001), indicating the need for further research to confirm these results.

## 1. Introduction

Medication-related osteonecrosis of the jaw (MRONJ) is a rare and debilitating multifactorial condition often associated with antiresorptive and antiangiogenic agents, used in the treatment of bone conditions and complications, such as osteoporosis, Paget’s disease, hypercalcemia, and metabolic bone lesions associated with malignancy [1,2,3].

According to the American Association of Oral and Maxillofacial Surgeons (AAOMS), the diagnosis of MRONJ requires the presence of three criteria simultaneously: current or previous treatment with antiresorptive or antiangiogenic agents, presence of visible or probe-able exposed bone in the maxillofacial region for more than eight weeks, and absence of a history of radiotherapy or metastasis in the jaws [1,2,4,5,6].

The AAOMS recognizes three classes of medications associated with MRONJ: bisphosphonates, RANKL ligand inhibitors, and antiangiogenic medications [1,2]. Bisphosphonates, derived from inorganic pyrophosphates, inhibit bone mineralization and resorption, promoting the apoptosis of osteoclasts [7]. Denosumab, a monoclonal antibody, binds to RANKL, inhibiting the formation and function of osteoclasts [6]. Although through different antiresorptive mechanisms, both drugs cause a decrease in the rate of bone turnover, leading to the accumulation of unresorbed hypermineralized bone [8]. In turn, antiangiogenic drugs inhibit the formation of new blood vessels, possibly leading to ischemia and hypoperfusion [6].

In 2003, Marx et al. reported the first cases of osteonecrosis of the jaws related to the use of bisphosphonates, and osteonecrosis of the jaw was subsequently recognized as an adverse effect of bisphosphonate treatment [2,6,7,9,10]. In 2010, Aghaloo et al. reported cases of osteonecrosis of the jaws related to the use of denosumab. Following this and other similar reports, and in order to include all the drugs implicated in osteonecrosis of the jaws, the AAOMS proposed changing the nomenclature from “Bisphosphonate-related osteonecrosis of the jaw” (BRONJ) to “Medication-related osteonecrosis of the jaw” (MRONJ) [2,6,7,9,10].

According to the AAOMS, the diagnosis of MRONJ is mostly clinical; however, the support of complementary diagnostic exams is advantageous in the diagnosis and evaluation of the progression of the disease, namely orthopantomography, computed tomography, and magnetic resonance imaging [8].

When it comes to recognized risk factors of MRONJ, antiresorptive medications such as bisphosphonates or denosumab and antiangiogenic medications such as aflibercept and sunitinib are associated with an increased risk of developing MRONJ [11]. It is necessary to evaluate the route of administration and duration of the treatment, with the risk being considered dose- and time-dependent, that is, as the dose increases over a longer period, the risk of developing osteonecrosis increases [2,11]. Several studies report that, among patients diagnosed with MRONJ, tooth extraction was a predisposing event in 62% to 82% of cases [11]. In turn, the presence of pre-existing periodontal or periapical infection in patients treated with antiresorptive or antiangiogenic medications is also a recognized risk factor for the development of MRONJ [2,11]. Finally, the development of MRONJ is more common in the mandible (75%) than in the maxilla (25%) and can occur in both jaws (4.5%) [2,11].

The AAOMS introduced a staging system in 2009, updated in 2022, to characterize the clinical presentation of MRONJ: patients at risk of developing MRONJ, stage 0, stage 1, stage 2, and stage 3 [5,8,11]. Patients at risk are asymptomatic and are undergoing treatment, or have been previously treated, with antiresorptive or antiangiogenic drugs, without evidence of necrotic bone. In stage 0, there is no evidence of necrotic bone, but patients present with nonspecific symptoms and clinical and imaging findings. In stage 1, it is possible to observe exposed and necrotic bone, or bone that can be probed through a fistula, in asymptomatic patients with no evidence of infection or inflammation, and it is also possible to observe radiographic findings characteristic of stage 0. In stage 2, it is possible to observe exposed and necrotic bone, or bone that can be probed through a fistula, in symptomatic patients, with evidence of infection or inflammation, and it is also possible to observe radiographic findings characteristic of stage 0. In stage 3, patients present exposed and necrotic bone, or bone capable of being probed through a fistula, with evidence of infection and at least one of the following signs: exposed necrotic bone extending beyond the region of the alveolar bone (lower border and ramus of the mandible, maxillary sinus, or zygomatic bone), pathological fracture, extraoral fistula, oroantral communication, and osteolysis extending to the lower border of the mandible or floor of the maxillary sinus [5,8,11].

The treatment of MRONJ is challenging and there is no consensus regarding the most appropriate management strategy [1,12]. According to the AAOMS, the main goals of treatment are to eliminate pain, control infection, and minimize the occurrence or progression of osteonecrosis [12]. The choice between surgical and non-surgical therapy must be specific and adapted to the needs of each patient. Nonsurgical strategies, such as administration of oral antiseptics and antibiotics, are useful, especially in patients with comorbidities that preclude surgery. Surgical therapy, including sequestrectomy, surgical debridement, and resective surgery, is a viable option, with high success rates [11].

For stage 0, management focuses on conservative care: educating the patient on oral hygiene, using symptomatic treatments like analgesics and antibiotics if needed, and applying chlorhexidine 0.12% rinses. Invasive dental procedures should be avoided to prevent disease progression. In stage 1, conservative management is recommended, including the use of a chlorhexidine rinse and regular follow-ups every 8–12 weeks. Systemic antibiotics are not typically needed unless infection occurs. Emerging treatments like low-level laser therapy (LLLT) may help with pain and inflammation. For stage 2, treatment recommendations include antibiotics (amoxicillin/clavulanate or clindamycin), chlorhexidine rinses, and analgesics. Limited debridement is one of the recommendations, but extensive surgery should be avoided. Hyperbaric oxygen therapy (HBOT) might aid healing, though its effectiveness is still debated. The most recent recommendations for stage 3 include broad-spectrum antibiotics, extensive surgical debridement or resection, and possible jaw reconstruction with flaps or grafts. Chlorhexidine rinses and intensive pain management along with infection control are critical. Adjunctive therapies like Leukocyte- and Platelet-Rich Fibrin (L-PRF) or Bone Morphogenetic Proteins (BMPs) may support healing, though evidence for these is mixed [11].

There is not yet sufficient evidence to recommend adjuvant therapies, namely L-PRF membranes, hyperbaric oxygen therapy, ozone therapy, use of vitamin E, pentoxifylline, and teriparatide, which is why these should not be recommended as the basis of MRONJ treatment. A multidisciplinary approach is essential, and collaboration between oncologists, rheumatologists, dentists, and oral and maxillofacial surgeons is essential for a comprehensive evaluation of the patient diagnosed with MRONJ [11].

The clinical practice of antiresorptive drug holidays to mitigate MRONJ risk in patients undergoing dentoalveolar surgery remains controversial. The historical use of a drug holiday was intended to decrease the prevalence of MRONJ subsequent to the performance of high-risk surgical procedures. The concern regarding this practice is the loss of efficacy of antiresorptive therapy with the development of skeletal-related events and fragility fractures. Factors for consideration may include disease-related risk (cancer vs. osteoporosis), drug-dosing frequency, duration of therapy, comorbidities, other medications (especially chemotherapy, steroids, or antiangiogenics), degree of underlying infection/inflammation, and extent of surgery to be performed. A special concern should be considered for suspending RANKL inhibitors in osteoporosis patients [11].

Several studies have demonstrated a rebound increase in bone resorption following the discontinuation of denosumab, resulting in an increased risk of multilevel vertebral fractures. If demosumab is to be suspended, the timing and duration of the holiday should be optimized in order to minimize this risk. The planned dentoalveolar surgery can be completed 3–4 months following the last dose of denosumab when the level of osteoclast inhibition is waning. It can then be reinstituted 6–8 weeks post-surgery. This management strategy minimizes the length of the drug holiday while maintaining a favorable environment for bone healing [11].

Actually, there is insufficient evidence to conclude that the use of the other interventions investigated would reduce the risk of MRONJ or would improve the healing of MRONJ [13].

## 2. Materials and Methods

This systematic review was carried out, according to the PRISMA (Preferred Reporting Items for Systematic Reviews and Meta-analyses) recommendations [14].

### 2.1. Search Strategy

An electronic search for case reports and case series was carried out, until March 2024, in the PubMed-Medline database, using the keywords “medication” AND “related” AND “osteonecrosis” AND “jaw”. The search was limited to human studies and articles written in the English language, and no time limit was set.

The following search was carried out: (“medication” AND “related” AND “osteonecrosis” AND “jaw”) AND (“Case Reports”[Publication Type] OR “Case Series”[Publication Type]) AND (“english”[Language]).

### 2.2. Selection Criteria

Reports and case series reported in the literature on medication-related osteonecrosis of the jaw were selected.

Letters to the editor, systematic or literature reviews, correspondence, and book chapters were excluded. Articles published in a language other than English and animal studies and articles whose text was not fully available were also excluded.

All cases that presented factors that could alter the immune response and, therefore, affect the healing process were also excluded in order to evaluate solely the influence of the medication on the development of osteonecrosis, namely poorly controlled diabetes mellitus (or without evidence of being controlled), rheumatoid arthritis, multiple myeloma, psoriatic arthritis, osteoarthritis, toxiphilic, alcoholic and smoking habits, and ingestion of corticosteroids and/or immunosuppressants [3,8]. Finally, cases in which patients underwent head and neck radiotherapy were also excluded, in order to exclude the possibility of osteoradionecrosis of the jaw. Therefore, all cases that included possible etiological factors of osteonecrosis of the jaw other than the drugs under study were excluded.

The selection, reading, and analysis of the articles included in this systematic review were performed by one researcher (F.F.) and were carried out manually. Articles that raised questions about their inclusion were reviewed by a second investigator (A.M.).

### 2.3. Data Extraction

Information regarding the parameters under study was collected manually. The articles were read, and the respective information was, in the first step, recorded in a Microsoft Excel 365 MSO spreadsheet (version 2403).

### 2.4. Study Risk of Bias Assessment

In order to evaluate the quality of the articles, the evaluation forms of case reports and case series from Joanna Briggs Institute–University of Adelaide were used [15].

### 2.5. Data Collected

Information was collected regarding the sex and age of the patients, stage of the disease, medication associated with the development of MRONJ (active ingredient, route of administration, and time of administration), anatomical location of osteonecrosis, therapy of MRONJ (antibiotic, mouthwash, surgery, and other treatments), and, finally, follow-up time from the beginning of treatment for the disease until its cure. All data were entered into the Microsoft^®^ Excel^®^ program for Microsoft 365 MSO (version 2403).

### 2.6. Data Analysis

Statistical analysis was performed using the SPSS^®^ program, version 28.0 (IBM, Armonk, NY, USA), and included descriptive statistics measures (absolute and relative frequencies) and inferential statistics (Fisher’s exact test).

## 3. Results

From the Boolean search in the PubMed-Medline database, using the aforementioned keywords, 178 results were obtained. In total, 144 articles were selected based on reading of the title. In the articles whose titles raised doubts, the respective abstracts were read; thus, 10 articles were selected. The 154 articles then selected were analyzed by the reviewer, and only those articles that had the full text available and that met the selection criteria were included. Therefore, 88 articles were included in this systematic review, corresponding to 62 case reports and 26 case series (Appendix A).

**Figure 1:**
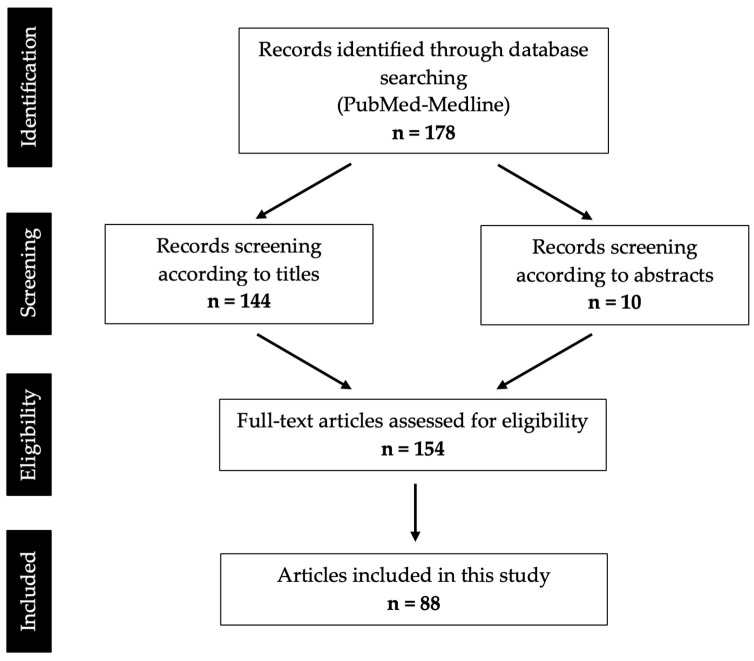
PRISMA flow diagram of search results and screening process.

Figure 1 represents the flowchart with the systematization of the selection process.

This systematic review included 151 cases of MRONJ for study. Not all articles included information on all the defined variables. For each variable, the actual number of cases providing the respective information is specified in the text.

### 3.1. Sex

**Figure 2:**
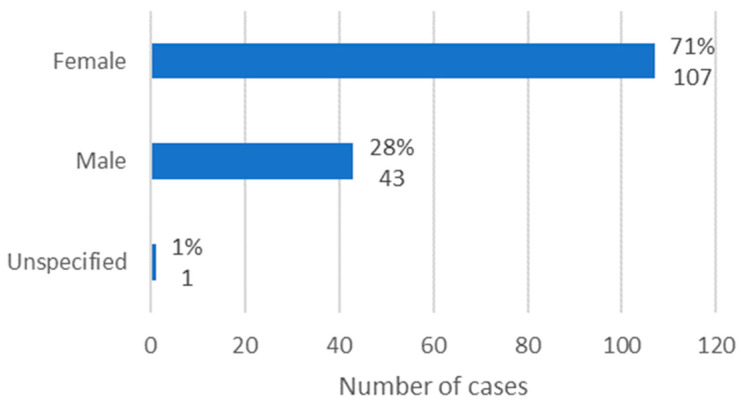
Distribution of the population by sex (relative frequency and absolute frequency).

Information regarding the sex of the patients was available in 150 cases. It was observed that females were the most affected, with 71% of cases (*n* = 107). In turn, males represented 28% of cases (*n* = 43), making a ratio of 2.49:1 (Figure 2).

### 3.2. Age

Information regarding age was available in 146 cases. The average age at diagnosis was 66.27 years, with a standard deviation of ±13.03. The minimum age was 23 years, and the maximum age was 89 years.

In relation to females, the average age was 67.23 years, with a standard deviation of ±13.07, corresponding to a minimum age of 27 years and a maximum age of 89 years. In males, the average age was 64.17 years (standard deviation ± 12.87), the minimum age was 23 years, and the maximum age was 82 years.

**Figure 3:**
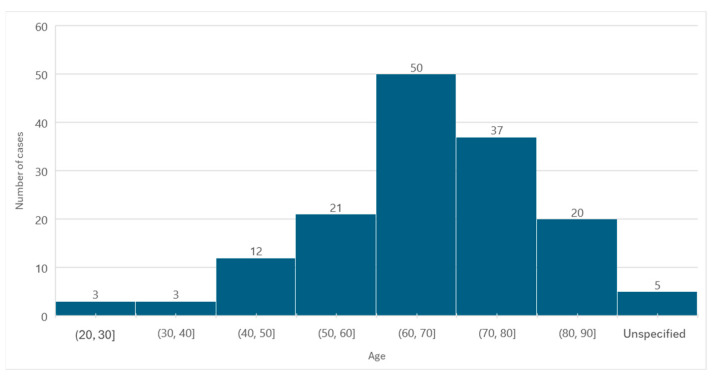
Distribution of the population by age (absolute frequency).

The age group with the highest number of MRONJ cases was 60 to 70 years old (*n* = 50) (Figure 3).

### 3.3. Stage

**Figure 4:**
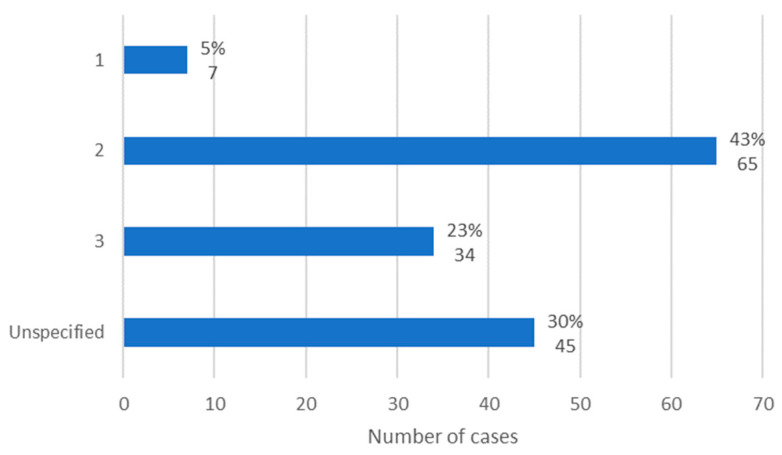
Distribution of the population by MRONJ stage (relative frequency and absolute frequency).

The MRONJ stage was specified in 106 cases, according to the staging system proposed by AAOMS. Stage 1 was verified in 5% of cases (*n* = 7), stage 2 in 43% of cases (*n* = 65), and stage 3 in 23% of cases (*n* = 34). Thus, it is concluded that in the studied population, stage 2 of MRONJ was the most observed (Figure 4).

### 3.4. Medication Associated with Osteonecrosis: Active Ingredient

**Figure 5:**
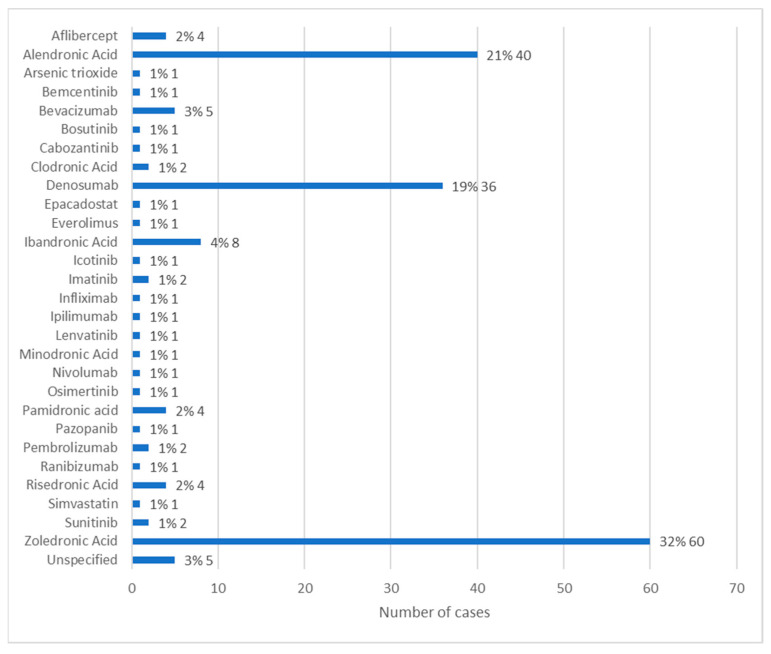
Distribution of the population by active ingredient administered (relative frequency and absolute frequency).

Information regarding the active ingredient associated with the development of MRONJ was available in 146 cases. The most used active ingredient was zoledronic acid, in 32% of cases (*n* = 60). The second most used active ingredient was alendronic acid, with 21% of cases (*n* = 40), followed by denosumab represented by 19% of cases (*n* = 36). Ibandronic acid was used in 4% of cases (*n* = 8) and bevacizumab in 3% of cases (*n* = 5). The other active ingredients each represent 1% of cases (*n* = 1 or *n* = 2). Of these, simvastatin and arsenic trioxide stand out, which, according to the literature, are not active ingredients commonly associated with MRONJ [16,17] (Figure 5).

It is important to highlight that the medications under study were not always used alone, with cases in which at least two active ingredients were administered to the same patient. In these cases, it was impossible to assertively determine whether just one of the active ingredients administered was associated with the development of MRONJ, or whether it was a combination of several.

### 3.5. Medication Associated with Osteonecrosis: Route of Administration

The route of administration of the active ingredients in question was available in 71 cases.

**Figure 6:**
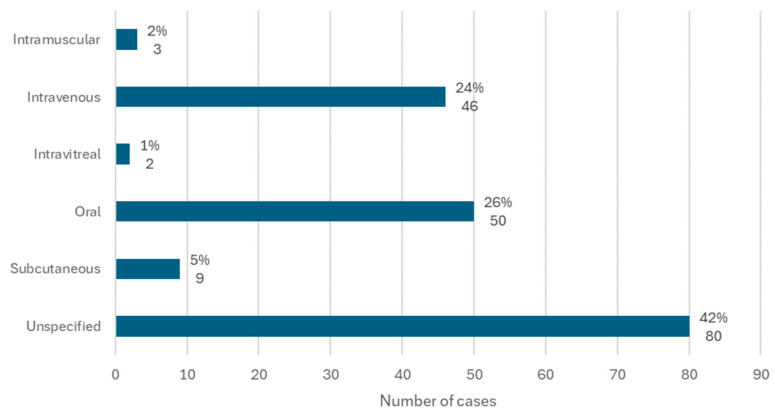
Distribution of the population by route of administration (relative frequency and absolute frequency).

The most common route of administration was oral, with 26% of cases (*n* = 50), followed by intravenous, with 24% of cases (*n* = 46). In 5% of cases (*n* = 9), the active ingredient was administered subcutaneously, and there were 2 cases in which the active ingredient associated with MRONJ was administered through intravitreal administration (Figure 6).

### 3.6. Medication Associated with Osteonecrosis: Administration Time

Information regarding the time between the first administration of the active ingredient and the diagnosis of MRONJ was available in 99 cases. The average administration time was 60.88 months, the minimum time passed was 1 month, and the maximum time was 252 months, with a standard deviation of ±50.92.

**Figure 7:**
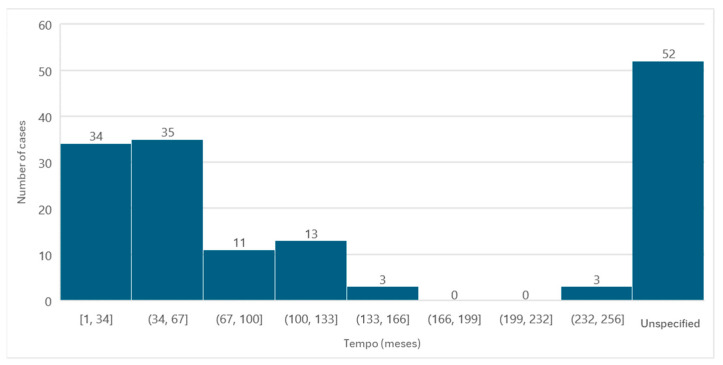
Distribution of the population by administration time (absolute frequency).

Of the cases in which the administration time was specified, it was observed that in a larger number of cases (*n* = 35), the administration time was between 34 and 67 months (Figure 7).

It should be noted that, in cases where more than one active ingredient that could be associated with the development of MRONJ was administered and which, therefore, had different administration intervals, only the first administration was considered, that is, the longest period of administration.

### 3.7. Anatomical Location

**Figure 8:**
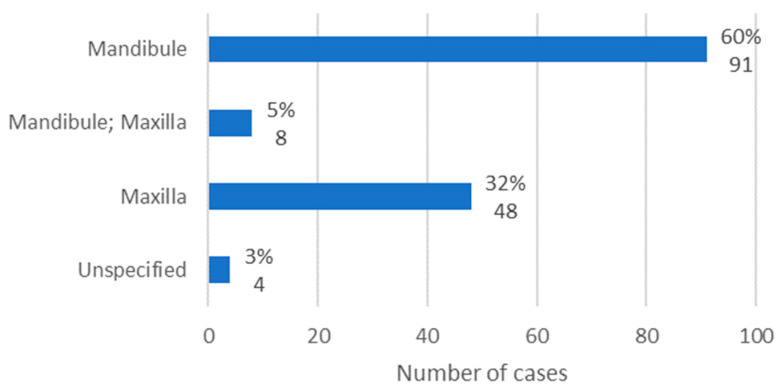
Population distribution by anatomical location of lesions (relative frequency and absolute frequency).

The anatomical location of osteonecrosis was available in 147 cases. The mandible was the most affected location, with 60% of cases (*n* = 91). In turn, the maxilla was affected in 32% of cases (*n* = 48). Finally, it was observed that, in only 5% of cases, both jaws were affected (Figure 8).

### 3.8. Targeted Treatment for Osteonecrosis (Antibiotics, Mouthwash, Surgery, and Others)

**Figure 9:**
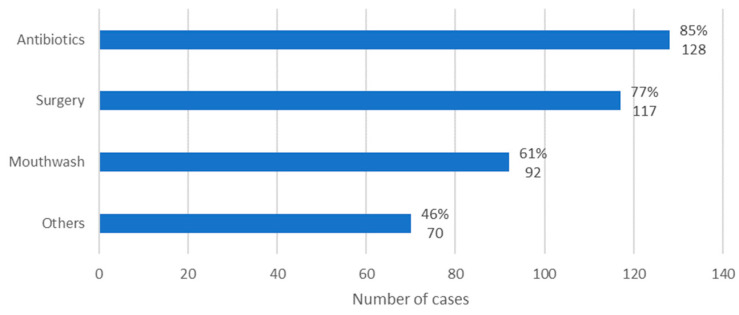
Distribution of the population by therapy instituted (relative frequency and absolute frequency).

In general, the MRONJ therapy instituted included administration of antibiotics, use of mouthwash, performance of surgical procedures, and use of unconventional treatments for MRONJ. In 85% of cases, antibiotics were administered (*n* = 128); in 77% of cases, surgical procedures were performed (*n* = 117); in 61% of cases, mouthwash was administered (*n* = 92); and, finally, in 46% of the cases, non-conventional treatments were carried out (*n* = 70) (Figure 9).

It is important to note that the types of treatments presented were used concomitantly in some cases, at least in combinations of two, and, in certain cases, all treatments presented were applied.

### 3.9. Targeted Treatment for Osteonecrosis: Antibiotics

**Figure 10:**
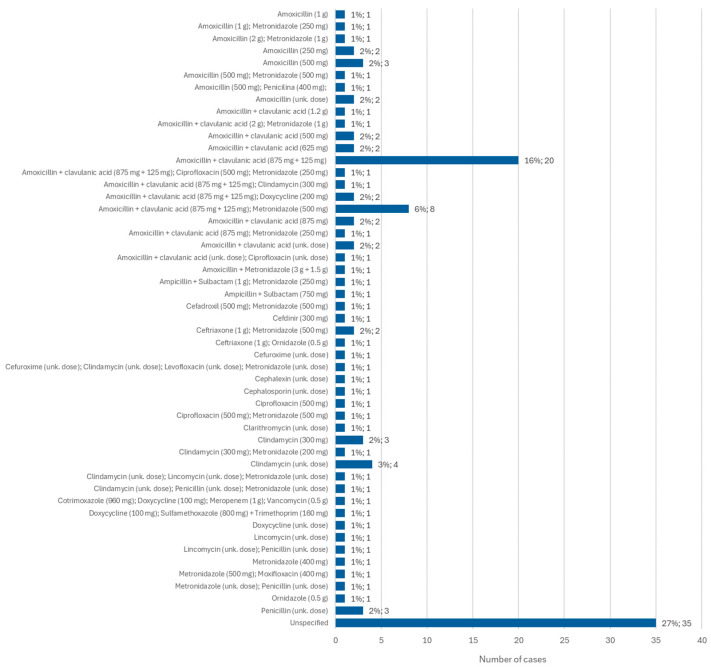
Distribution of the population by antibiotic administered—relative frequency and absolute frequency (unk. dose = unknown dose).

Information regarding the antibiotic administered for the treatment of MRONJ was available in 116 cases. Of these, amoxicillin + clavulanic acid (875 mg + 125 mg) was the most administered antibiotic, in 16% of cases (*n* = 20). It is followed by amoxicillin + clavulanic acid (875 mg + 125 mg) administered together with metronidazole (500 mg) in 6% of cases (*n* = 8). Clindamycin was administered, alone and at an unknown dose, in 3% of cases (*n* = 4) and at a dose of 300 mg, alone, in 2% of cases (*n* = 3) (Figure 10).

### 3.10. Targeted Treatment for Osteonecrosis: Mouthwash

**Figure 11:**
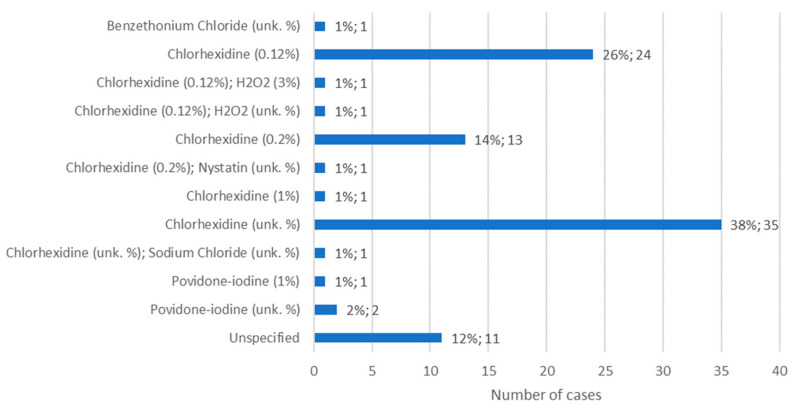
Distribution of the population by mouthwash administered—relative frequency and absolute frequency (unk. % = unknown concentration).

Information about the mouthwash administered for the treatment of MRONJ was available in 140 cases. The most commonly used mouthwash was chlorhexidine, although its concentration was not specified, in 38% of cases (*n* = 35). When the concentration was specified, it was observed that 0.12% chlorhexidine was used more than 0.2% chlorhexidine, representing 26% of cases (*n* = 24) and 14% of cases (*n* = 13), respectively. A concentration of 0.12% chlorhexidine was also administered together with hydrogen peroxide, at 3% or at an unknown concentration, in 1% of cases (*n* = 1) in both situations. In turn, 0.2% chlorhexidine was administered together with a nystatin solution, of unknown concentration, in 1% of cases (*n* = 1) (Figure 11).

### 3.11. Targeted Treatment for Osteonecrosis: Surgical Procedure

**Figure 12:**
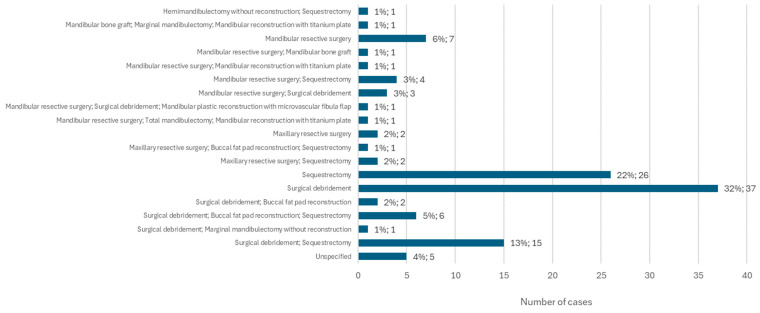
Distribution of the population by surgical procedure (relative frequency and absolute frequency).

The type of surgical procedure performed in the treatment of MRONJ was specified in 146 cases. Surgical debridement alone was the most common surgical procedure, accounting for 32% of cases (*n* = 37). Sequestrectomy follows, performed alone, in 22% of cases (*n* = 26). These two surgical procedures were also performed together in 13% of cases (*n* = 15). Mandibular resective surgery was the only surgical option chosen in 6% of cases (*n* = 7). This type of surgery was performed on the lower jaw together with sequestrectomy in 3% of cases (*n* = 4) and on the upper jaw, also together with sequestrectomy, in 2% of cases (*n* = 2) (Figure 12).

### 3.12. Other Treatments Aimed at Osteonecrosis

**Figure 13:**
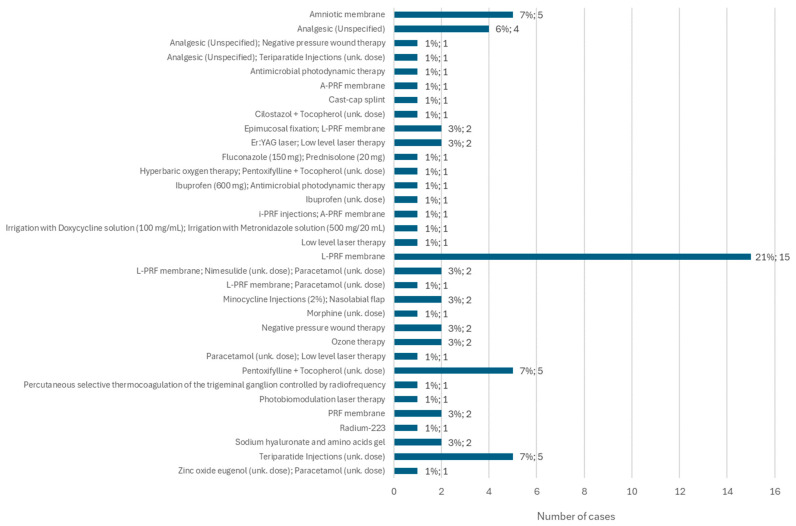
Distribution of the population by other treatments implemented (relative frequency and absolute frequency).

Among the different types of non-conventional treatments administered, the L-PRF membrane stands out, applied in 21% of cases (*n* = 15). Furthermore, teriparatide injections, at an unspecified dose, were administered in 7% of cases (*n* = 5). In the same percentage of the population, a combination of pentoxifylline + tocopherol was administered, in unknown doses, and amniotic membrane was applied independently. Of the other types of non-conventional treatment, ozone therapy and negative pressure wound therapy stand out, each applied separately in two cases (Figure 13).

### 3.13. Follow-Up Time from the Start of MRONJ Treatment until Its Cure

**Figure 14:**
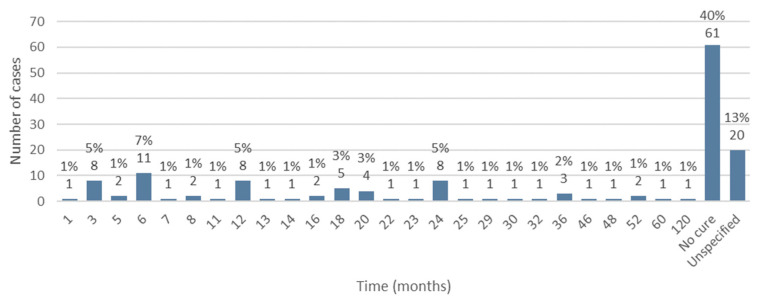
Distribution of the population by follow-up time (relative frequency and absolute frequency).

Information regarding the follow-up time from the beginning of implementation of MRONJ treatment until its cure was available in 131 cases. Of these, no cure for the disease was observed in 40% of the studied population (*n* = 61). The minimum follow-up time from the start of treatment to cure was 1 month and the maximum time was 120 months. The average follow-up time was 18.56 months, with a standard deviation of ±18.13 (Figure 14).

### 3.14. Relationship between Established Therapy and Cure of MRONJ

It was possible to relate the type of treatment instituted (antibiotic, mouthwash, surgical procedure, and others) and the existence or non-existence of a cure for MRONJ, through the application of Fisher’s exact test, analyzing the exact two-sided significance value obtained. Therefore, it is concluded that, in cases where *p* ≤ 0.05, the relationship between the two variables is statistically significant.

Regarding the relationship between the administration of antibiotics and obtaining a cure, it is observed that, of the 128 cases in which antibiotics were administered, there was a cure in 74 cases (57.8%) (Table 1). The relationship between these two variables resulted in a value of *p* = 0.359 (>0.05), concluding that the relationship between the administration of antibiotics and the achievement of a cure is not statistically significant (Table 2).

From the relationship between the administration of mouthwash and the achievement of a cure, it can be seen that, of the 92 cases in which mouthwash was administered, a cure was achieved in 54 cases (58.7%) (Table 3). The application of Fisher’s test between the two variables mentioned resulted in a value of *p* = 0.865, concluding that the relationship between the administration of mouthwash and the achievement of a cure is not statistically significant (Table 4).

From the relationship between the application of surgical procedures and obtaining a cure, it is observed that, of the 117 cases in which surgery was performed, 72 were cured (61.5%) (Table 5). The application of Fisher’s test revealed a value of *p* = 0.429 (*p* > 0.05); therefore, the relationship between surgery and a cure is not statistically significant (Table 6).

Relating the performance of other treatments with obtaining a cure, it is observed that, of the 70 cases in which non-conventional treatments were applied, there was a cure in 52 cases (74.3%) (Table 7). The application of Fisher’s test revealed a value of *p* < 0.001; therefore, since *p* < 0.05, it is concluded that there is a statistically significant relationship between the application of unconventional treatments and the achievement of a cure (Table 8).

**Figure 15:**
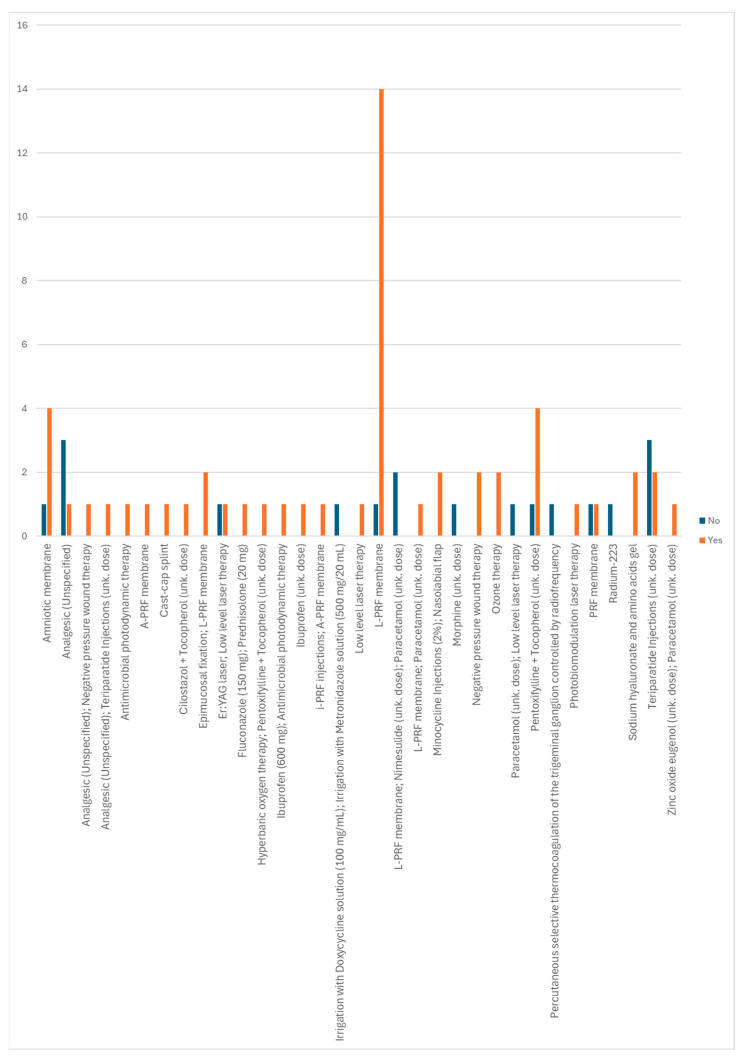
Distribution of non-conventional treatments according to whether or not a cure is obtained.

It is possible to observe, in Figure 15, which non-conventional treatments applied were associated with obtaining a cure. Of these, the L-PRF membrane, the amniotic membrane, and the pentoxifylline + tocopherol combination stand out, although there have also been fewer cases in which these treatments were not associated with curing the disease. Of the non-conventional treatments applied that only showed a cure, the association of epimucosal fixation and L-PRF membrane, the association of minocycline injections and a nasolabial flap, negative pressure wound therapy, ozone therapy, and sodium hyaluronate and amino acid gels stand out.

In Appendix B, it is noted that the systematization of the different therapies instituted and the respective result regarding the obtainment or not of a cure, in all cases, is included.

## 4. Discussion

According to the results obtained, it was observed that females were more affected than males in a ratio of 2.49:1. This result is in line with the literature, which reports a higher prevalence of MRONJ in females, probably reflecting the underlying disease for which the drugs are administered, such as osteoporosis and breast cancer, which are more common in women [6,11,18]. Preventing MRONJ in these high-risk groups composed of women requires a comprehensive approach. It involves recognizing the impact of antiresorptive therapies on bone healing, emphasizing the need for coordinated dental care, pretreatment management, and ongoing education for patients and healthcare professionals about MRONJ risks and prevention strategies [11].

The average age at diagnosis was 66.27 years, with the 60 to 70 years age group being the most affected. This is in line with the literature, which indicates that ages over 65 years represent a risk factor for MRONJ [7]. In a retrospective statistical study that analyzed 70 articles, the average age of patients was 62 years [19].

According to Ojha et al. [20], stages 1 and 2 of MRONJ are the most commonly observed. However, the results of two retrospective studies demonstrate that stage 2 was the most frequently diagnosed, one with 129 patients [21] and the other with 71 patients [22].

A retrospective study by Ahdi et al. [1], which analyzed cases of MRONJ reported in the FAERS database from 2010 to 2021, identified the medications most associated with the development of MRONJ. The 10 most frequently associated drugs were, in descending order, zoledronic acid, alendronic acid, denosumab, pamidronic acid, ibandronic acid, lenalidomide, risedronic acid, sunitinib, bevacizumab, and prednisolone. These findings corroborate the results of this review, where zoledronic acid was the most used drug, followed by alendronic acid and denosumab.

A clinical case describes the development of MRONJ in a patient with no history of administration of antiresorptive or antiangiogenic drugs, corticosteroids, or head and neck radiotherapy, medicated only with simvastatin for hypercholesterolemia [16]. This case highlights the need to develop more studies on the influence of different drugs on the oral cavity.

The literature suggests a higher risk of MRONJ in patients with malignant tumors treated with intravenous bisphosphonates compared to patients treated with oral bisphosphonates for osteoporosis [2,5,8,23]. However, the results of this review show that the most common route of administration of medications was oral (26% of cases), followed by intravenous (24%). This allows us to question the premise that there is a greater risk of developing MRONJ with intravenous medications, highlighting the need to pay equal attention to oral administration of this type of medication.

The duration of treatment with antiresorptive or antiangiogenic agents until the diagnosis of MRONJ varied significantly, with the largest number of cases falling within the 34- to 67-month administration interval. A literature review that included 50 patients demonstrated an average treatment duration of 48.68 months [24]. In the present review, the average duration of treatment was 60.88 months, with a minimum time of 1 month and a maximum time of 252 months. These data, together with the fact that, in the aforementioned literature review, the majority of patients were in an advanced stage of MRONJ at the time of diagnosis, emphasize the importance of rigorous monitoring of patients medicated with antiresorptive and antiangiogenic agents in order to detect early signs of the disease.

According to Ruggiero et al. [11], MRONJ lesions affect the mandible (75%) more than the maxilla (25%) and may involve both jaws (4.5%). Thus, the literature corroborates the results obtained, in which the mandible was the most affected location (60%), followed by the maxilla (32%) and both jaws (5%).

For the conservative treatment of MRONJ, 0.12% or 0.2% chlorhexidine gluconate represents an efficient topical bacteriostatic–bactericidal agent that works to reduce the oral bacterial population, including biofilms that promote infection [19]. In fact, according to the results obtained, the most used mouthwash was chlorhexidine, and when the information was specified, the most used concentration was 0.12%. However, oral antibiotics are the most important agents to treat the infection in osteonecrosis, and, since the infections associated with this disease are polymicrobial, broad-spectrum antibiotics are recommended, such as the combination of amoxicillin + clavulanic acid, ampicillin, metronidazole, or clindamycin [19]. This is in line with the results obtained, in which amoxicillin + clavulanic acid (875 mg + 125 mg) was the most administered antibiotic.

A systematic review by Seluki et al. [25] showed that the non-surgical approach may be useful in preventing disease progression in patients ineligible for surgery, but effective resolution of osteonecrosis should not be expected. The results of Fisher’s exact test revealed a value of *p* = 0.359 for the relationship between antibiotic administration and MRONJ cure and *p* = 0.865 for the relationship between mouthwash administration and cure, indicating that there is no relationship with a statistically significant difference between the treatments mentioned and the achievement of a cure for MRONJ.

The most common surgical procedures were surgical debridement (32%) and sequestrectomy (22%), performed alone. Although Nicolatou-Galitis et al. [3] suggest that surgical procedures are more effective in advanced cases of MRONJ, Fisher’s test showed a value of *p* = 0.429, indicating that the relationship between performing surgery and obtaining a cure for MRONJ is not statistically significant.

Carrying out unconventional treatments showed a statistically significant relationship with obtaining a cure for MRONJ (*p* < 0.001), despite the literature stating that the effectiveness of these treatments has not been proven [3]. These results suggest that non-conventional treatments may be an effective alternative for resolving MRONJ.

The relationship between carrying out conventional treatments alone and obtaining a cure was not statistically significant, particularly with regard to the administration of antibiotics, the use of mouthwash, and surgery. However, there is the possibility that combinations of conventional treatments may be effective in achieving a cure. To this end, it may be interesting to expand the study in the future in order to evaluate the statistical significance of the combinations of different treatments in obtaining a cure.

The literature regarding the time elapsed from the start of treatment for MRONJ until a cure is achieved (follow-up) is scarce and significantly heterogeneous. Mourão et al. [26] reported a mean follow-up time of 23.5 months, while a meta-analysis demonstrated a mean follow-up time of 8.7 months [27]. In the present review, the average follow-up was 18.56 months, with a minimum of 1 month and a maximum of 120 months.

Dentists should ensure thorough pretreatment dental assessments and complete any necessary invasive procedures before initiating antiresorptive or antiangiogenic therapy to reduce MRONJ risk. During therapy, they should prioritize non-invasive dental treatments, carefully assess the risks and benefits of any invasive procedures, and, in consultation with the prescribing physician, consider a temporary discontinuation of the medication. Additionally, regular dental check-ups and patient education on maintaining excellent oral hygiene and recognizing MRONJ symptoms are essential for effective prevention and early detection [11].

This systematic review highlights important findings but also underscores the limitations inherent in the current body of evidence, primarily consisting of case reports and clinical case series. These sources, while valuable, are limited by factors such as lack of randomization, variability in reports, and absence of control groups. Additionally, publication bias may skew the results toward more positive outcomes. To enhance the robustness and generalizability of the findings, there is a clear need for more well-designed, randomized clinical trials. Such studies are crucial to provide more definitive insights into the efficacy and success of treatments, ultimately guiding more effective clinical practices.

## 5. Conclusions

MRONJ is a complex condition, the pathophysiology of which is not yet fully understood. It is mainly associated with the use of antiresorptive and antiangiogenic drugs, but cases of MRONJ associated with other classes of drugs have been described, highlighting the need to develop more studies to clarify the pathophysiology of the disease and, thus, improve patients’ quality of life.

Based on the relationship between the type of treatment instituted and the achievement of a cure, the only statistical significance found was related to non-conventional treatments, which allows us to formulate the hypothesis that these treatments, carried out alone or associated with conservative or surgical therapies, can be effective in resolving MRONJ.

The treatment of MRONJ remains challenging, due to the lack of consensus among different authors regarding the most appropriate approach protocol and the variation in patients’ response to established therapeutic interventions, meaning that treatment must be individualized and adapted to clinical needs.

## Figures and Tables

**Table 1:** Count in relation to the relationship between two variables (antibiotic administration and obtaining a cure).

			Cure		Total
			No	Yes	
Antibiotics	No	Count	7	16	23
		% within Antibiotics	30.4%	69.6%	100.0%
		% within Cure	11.5%	17.8%	15.2%
		% of Total	4.6%	10.6%	15.2%
	Yes	Count	54	74	128
		% within Antibiotics	42.2%	57.8%	100.0%
		% within Cure	88.5%	82.2%	84.8%
		% of Total	35.8%	49.0%	84.8%
Total		Count	61	90	151
		% within Antibiotics	40.4%	59.6%	100.0%
		% within Cure	100.0%	100.0%	100.0%
		% of Total	40.4%	59.6%	100.0%

**Table 2:** Application of Fisher’s test in the relationship between two variables (administration of antibiotics and achievement of cure).

	Value	df	Asymptotic Significance (2-Sided)	Exact Sig. (2-Sided)	Exact Sig. (1-Sided)
Pearson Chi-Square	1.118 ^a^	1	0.290		
Continuity Correction ^b^	0.684	1	0.408		
Likelihood Ratio	1.151	1	0.283		
Fisher’s Exact Test				0.359	0.205
*N* of Valid Cases	151				

^a^ 0 cells (0.0%) have expected count less than 5. The minimum expected count is 9.29. ^b^ Computed only for a 2 × 2 table.

**Table 3:** Count in relation to the relationship between two variables (mouthwash administration and obtaining a cure).

			Cure		Total
			No	Yes	
Mouthwash	No	Count	23	36	59
		% within Mouthwash	39.0%	61.0%	100.0%
		% within Cure	37.7%	40.0%	39.1%
		% of Total	15.2%	23.8%	39.1%
	Yes	Count	38	54	92
		% within Mouthwash	41.3%	58.7%	100.0%
		% within Cure	62.3%	60.0%	60.9%
		% of Total	25.2%	35.8%	60.9%
Total		Count	61	90	151
		% within Mouthwash	40.4%	59.6%	100.0%
		% within Cure	100.0%	100.0%	100.0%
		% of Total	40.4%	59.6%	100.0%

**Table 4:** Application of Fisher’s test in the relationship between two variables (administration of mouthwash and achievement of a cure).

	Value	df	Asymptotic Significance (2-Sided)	Exact Sig. (2-Sided)	Exact Sig. (1-Sided)
Pearson Chi-Square	0.080 ^a^	1	0.777		
Continuity Correction ^b^	0.013	1	0.909		
Likelihood Ratio	0.081	1	0.777		
Fisher’s Exact Test				0.865	0.456
*N* of Valid Cases	151				

^a^ 0 cells (0.0%) have expected count less than 5. The minimum expected count is 23.83. ^b^ Computed only for a 2 × 2 table.

**Table 5:** Count in relation to the relationship between two variables (surgical procedures and obtaining a cure).

			Cure		Total
			No	Yes	
Surgery	No	Count	16	18	34
		% within Surgery	47.1%	52.9%	100.0%
		% within Cure	26.2%	20.0%	22.5%
		% of Total	10.6%	11.9%	22.5%
	Yes	Count	45	72	117
		% within Surgery	38.5%	61.5%	100.0%
		% within Cure	73.8%	80.0%	77.5%
		% of Total	29.8%	47.7%	77.5%
Total		Count	61	90	151
		% within Surgery	40.4%	59.6%	100.0%
		% within Cure	100.0%	100.0%	100.0%
		% of Total	40.4%	59.6%	100.0%

**Table 6:** Application of Fisher’s test in the relationship between two variables (surgical procedures and achievement of a cure).

	Value	df	Asymptotic Significance (2-Sided)	Exact Sig. (2-Sided)	Exact Sig. (1-Sided)
Pearson Chi-Square	0.809 ^a^	1	0.369		
Continuity Correction ^b^	0.491	1	0.483		
Likelihood Ratio	0.801	1	0.371		
Fisher’s Exact Test				0.429	0.241
N of Valid Cases	151				

^a^ 0 cells (0.0%) have expected count less than 5. The minimum expected count is 13.74. ^b^ Computed only for a 2 × 2 table.

**Table 7:** Count in relation to the relationship between two variables (other treatments and obtaining a cure).

			Cure		Total
			No	Yes	
Others	No	Count	43	38	81
		% within Others	53.1%	46.9%	100.0%
		% within Cure	70.5%	42.2%	53.6%
		% of Total	28.5%	25.2%	53.6%
	Yes	Count	18	52	70
		% within Others	25.7%	74.3%	100.0%
		% within Cure	29.5%	57.8%	46.4%
		% of Total	11.9%	34.4%	46.4%
Total		Count	61	90	151
		% within Others	40.4%	59.6%	100.0%
		% within Cure	100.0%	100.0%	100.0%
		% of Total	40.4%	59.6%	100.0%

**Table 8:** Application of Fisher’s test in the relationship between two variables (other treatments and achievement of a cure).

	Value	df	Asymptotic Significance (2-Sided)	Exact Sig. (2-Sided)	Exact Sig. (1-Sided)
Pearson Chi-Square	11.684 ^a^	1	<0.001		
Continuity Correction ^b^	10.575	1	0.001		
Likelihood Ratio	11.939	1	<0.001		
Fisher’s Exact Test				<0.001	<0.001
*N* of Valid Cases	151				

^a^ 0 cells (0.0%) have expected count less than 5. The minimum expected count is 28.28. ^b^ Computed only for a 2 × 2 table.

## Appendix A

**Table A1:** Summary of the articles included in the systematic review (CS = case series; CR = case report).

Type of Study	Year	Author(s)	Article Title	Ref.
CS	2018	A Khominsky; MAWT Lim	“Spontaneous” medication-related osteonecrosis of the jaw; two case reports and a systematic review	[28]
CR	2023	Abrar A. Alamoudi; Axel Ruprecht; Anita Gohel; Joseph Katz	Maxillary Sinusitis Induced by Medication-Related Osteonecrosis of the Jaw	[29]
CR	2020	Agostino Guida; Francesco Perri; Franco Ionna; Paolo A. Ascierto; Antonio M. Grimaldi	New-generation anticancer drugs and medication-related osteonecrosis of the jaw (MRONJ): Late onset 3 years after ipilimumab endovenous administration with a possible role of target therapy	[30]
CR	2022	Alastair N Goss	Osteonecrosis of the jaw and denosumab	[31]
CS	2020	Alessandra Raimondi; Noemi Simeone; Marco Guzzo; Massimo Maniezzo; Paola Collini; Carlo Morosi; Francesca Gabriella Greco; Anna Maria Frezza; Paolo G Casali; Silvia Stacchiotti	Rechallenge of denosumab in jaw osteonecrosis of patients with unresectable giant cell tumour of bone: a case series analysis and literature review	[32]
CR	2020	Amanda Azevedo Torres; Beatriz Leal de Freitas; Patrick Parry Carneiro; André Luca Araujo de Sousa; Maria Ângela Arêa Leão Ferraz; Jean de Pinho Mendes; André Luiz Ferreira Costa; Antonione Santos Bezerra Pinto	Medication-Related Osteonecrosis of the Jaw and Low-Level Laser Therapy as Adjuvant Treatment: A Case Report	[33]
CR	2021	Amerigo Giudice; Alessandro Antonelli; Danila Muraca; Leonzio Fortunato	Usefulness of Advanced-Platelet Rich Fibrin (A-PRF) and Injectable-Platelet Rich Fibrin (i-PRF) in the Management of a Massive Medication-Related Osteonecrosis of the Jaw (MRONJ): A 5-Years Downloaded Follow-Up Case Report	[34]
CR	2019	Ana Karina Sarmiento L	Resolution without surgery of an advanced stage of medication-related osteonecrosis of the jaw (MRONJ) in a patient who could not suspend her treatment for osteoporosis	[35]
CS	2021	Antonio Cortese; Antonio Casarella; Candace M. Howard; Pier Paolo Claudio	Epi-Mucosa Fixation and Autologous Platelet-Rich Fibrin Treatment in Medication-Related Osteonecrosis of the Jaw	[36]
CR	2016	Arvind Muthukrishnan; Laliytha Bijai Kumar; Gomathi Ramalingam	Medication-related osteonecrosis of the jaw: a dentist’s nightmare	[37]
CR	2018	Caroline Ballardin; Cecilia Luiz Pereira-Stabile; Glaykon Alex Vitti Stabile	Use of a generic violet light in the surgical management of medication-related osteonecrosis of the jaws: a technical note	[38]
CS	2015	Mattia Berrone; Filippo Umberto Florindi; Vincenzo Carbone; Carola Aldiano; Monica Pentenero	Stage 3 Medication-Related Osteonecrosis of the Posterior Maxilla: Surgical Treatment Using a Pedicled Buccal Fat Pad Flap: Case Reports	[39]
CS	2015	Cameron Y. S. Lee; Jon B. Suzuki	Medication-Related Osteonecrosis of the Jaws From Once Per Year Intravenous Zoledronic Acid (Reclast): Report of 4 Cases	[40]
CS	2022	F Gülfeşan Çanakçi; Nilay Er; Gonca Duygu; G Füsun Varol	Surgical management of stage-2 medication-related osteonecrosis of the jaw with transplantation of human amniotic membrane: Preliminary results	[41]
CS	2019	Carlos Fernando de Almeida Barros Mourão; Mônica Diuana Calasans-Maia; Massimo Del Fabbro; Fabrício Le Drapper Vieira; Rafael Coutinho de Mello Machado; Raphaela Capella; Richard J Miron; Gutemberg Gomes Alves	The use of Platelet-rich Fibrin in the management of medication-related osteonecrosis of the jaw: a case series	[26]
CR	2020	Caspar V. Bumm; Matthias Folwaczny; Uta C. Wölfle	Necrotizing periodontitis or medication-related osteonecrosis of the jaw (MRONJ) in a patient receiving Bemcentinib—a case report	[42]
CR	2023	Yu-Feng Chen; Hong-Po Chang	Medication-Related Osteonecrosis of the Jaw	[43]
CS	2019	Christos Yapijakis; Veronica Papakosta; Stavros Vassiliou	ACE Gene Variant Causing High Blood Pressure May Be Associated With Medication-related Jaw Osteonecrosis	[44]
CR	2023	Ivan José Correia-Neto; Ana Carolina Evangelista Colafemina; Isabel Schausltz Pereira Faustino; Alan Roger Santos-Silva; Pablo Agustin Vargas; Márcio Ajudarte Lopes	Medication-related osteonecrosis in torus palatinus: Report of a case and literature review	[45]
CR	2019	Renato Patrizio Costa; Vincenzo Tripoli; Alessandro Princiotta; Alessandra Murabito; Maria Licari; Rodolfo Mauceri; Giuseppina Campisi; Antonio Pinto	Can radium 223 be a conservative non-surgical management of medication-related osteonecrosis of the jaw?	[46]
CR	2017	Enrico Nastro Siniscalchi; Alessandro Allegra; Francesco Saverio De Ponte; Giacomo Oteri; Gabriele Cervino; Floriana Lauritano; Caterina Musolino; Marco Cicciù	Spontaneous Healing of Clodronate-Related Osteonecrosis of the Jaw	[47]
CS	2021	Erica Vettori; Giulia Pipinato; Rossana Bussani; Fulvia Costantinides; Vanessa Nicolin; Lorenzo Bevilacqua; Michele Maglione	Therapeutic Approach in the Treatment of Medication-Related Osteonecrosis of the Jaw: Case Series of 3 Patients and State of the Art on Surgical Strategies	[48]
CS	2022	Erofili Papadopoulou; Emmanouil Vardas; Styliani Tziveleka; Maria Georgaki; Maria Kouri; Konstantinos Katoumas; Evangelia Piperi; Nikolaos G. Nikitakis	Oral Side Effects in Patients with Metastatic Renal Cell Carcinoma Receiving the Antiangiogenic Agent Pazopanib—Report of Three Cases	[49]
CR	2017	Eunae Sandra Cho; Seung Wook Jung; Hwi-Dong Jung; In Yong Lee; Tai-Soon Yong; Su Jin Jeong; Hyun Sil Kim	A Case of Pentastomiasis at the Left Maxilla Bone in a Patient with Thyroid Cancer	[50]
CR	2020	F. Bennardo; C. Buffone; D. Muraca; A. Antonelli; A. Giudice	Medication-Related Osteonecrosis of the Jaw with Spontaneous Hemimaxilla Exfoliation: Report of a Case in Metastatic Renal Cancer Patient under Multidrug Therapy	[51]
CR	2021	Façanha de Carvalho E; Maitê Bertotti; Cesar Augusto Migliorati; Andre Caroli Rocha	Cilostazol and Tocopherol in the Management ofMedication Related Osteonecrosis of the Jaw: NewInsights From a Case Report	[52]
CR	2022	Feng Wang; Shengnan Wei; Zexuan Zhang; Yuan Zhang; Jingya He; Bin Sun	Osimertinib: Another medication related to osteonecrosis of the jaws? A case report and literature review	[53]
CR	2015	Fernandez Ayora A; Herion F; Rompen E; Reginster JY; Magremanne M; Lambert F	Dramatic osteonecrosis of the jaw associated with oral bisphosphonates, periodontitis, and dental implant removal	[54]
CR	2016	G. Markose; R.M. Graham	Cast-cap splint in the management of medication-related osteonecrosis of the jaw	[55]
CR	2020	George Táccio de Miranda Candeiro; Vivian Bradaschia-Correa; Silvana Cristina Gama Vaz; Fabrício Bitu Sousa; Rafael Linard Avelar; Giulio Gavini; Cyrene Piazera Silva Costa; Ceci Nunes Carvalho	Spontaneous Bisphosphonate-related Osteonecrosis Associated with a Tooth that Had a Necrotic Pulp: A Case Report	[56]
CR	2021	George-Adrian Ciobanu; Mircea Ionuţ Gheorghiţă; Sanda Mihaela Popescu; Ionela Elisabeta Staicu Octavian Mihnea Petrescu	Mandibulectomy Reconstruction with Pectoralis Major Island Flap Associated with Primary Reconstruction Plate for Mandibular Medication-Related Osteonecrosis	[57]
CR	2017	Gianfranco Favia; Angela Tempesta; Luisa Limongelli; Vito Crincoli; Florenzo Iannone; Giovanni Lapadula; Eugenio Maiorano	A Case of Osteonecrosis of the Jaw in a Patient with Crohn’s Disease Treated with Infliximab	[58]
CR	2015	Gianfranco Favia; Angela Tempesta; Luisa Limongelli; Vito Crincoli; Adriano Piattelli; Eugenio Maiorano	Metastatic Breast Cancer in Medication-Related Osteonecrosis Around Mandibular Implants	[59]
CR	2017	Gustavo Antonio Correa Momesso; Fábio Roberto de Souza Batista; Cecília Alves de Sousa; Valthierre Nunes de Lima; Tárik Ocon Braga Polo; Jaqueline Suemi Hassumi; Idelmo Rangel Garcia Júnior; Leonardo Perez Faverani	Successful Use of Lower-Level Laser Therapy in the Treatment of Medication-Related Osteonecrosis of the Jaw	[60]
CS	2016	Gustavo Maluf; Milena Correia de Pinho; Sandra Ribeiro de Barros da Cunha; Paulo Sérgio da Silva Santos; Eduardo Rodrigues Fregnani	Surgery Combined with LPRF in Denosumab Osteonecrosis of the Jaw: Case Report	[61]
CS	2017	Gustavo Maluf; Rogério Jardim Caldas; Paulo Sérgio Silva Santos	The use of leukocyte-and platelet-rich fibrin (LPRF) in the treatment of medication related osteonecrosis of the jaws (MRONJ)	[62]
CS	2016	Hani Mawardi; Peter Enzinger; Nadine McCleary; Reshma Manon; Alessandro Villa; Nathaniel Treister; Sook-Bin Woo	Osteonecrosis of the jaw associated with ziv-aflibercept	[63]
CS	2022	Lei Hao; Zhuoli Tian; Shanli Li; Kemin Yan; Yan Xue	Osteonecrosis of the jaw induced by bisphosphonates therapy in bone metastases patient: Case report and literature review	[64]
CS	2014	Sebastian Hoefert; Martin Grimm; Feraydoon Sharghi; Andreas Geist; Michael Krimmel; Siegmar Reinert	Atraumatic tooth extraction in patients taking bisphosphonates: a review of literature and experience with three cases	[65]
CS	2010	Sebastian Hoefert; Harald Eufinger	Sunitinib may raise the risk of bisphosphonate-related osteonecrosis of the jaw: presentation of three cases	[66]
CR	2020	I. Oz; I. Kaplan; S. Kleinman; S. Arbel; A. Shuster	Medication-related osteonecrosis of the jaws associated with intravitreal administration of ranibizumab	[67]
CR	2016	Ilaria Giovannacci; Marco Meleti; Domenico Corradi; Paolo Vescovi	Clinical Differences in Autofluorescence Between Viable and Nonvital Bone: A Case Report With Histopathologic Evaluation Performed on Medication-Related Osteonecrosis of the Jaws	[68]
CR	2022	Injamamul L. Niloy; Jason N. Burkes	The Role of Endotracheal Tube in Medication-Related Osteonecrosis of the Jaw—A Case Report	[69]
CR	2020	J. Decaux; M. Magremanne	Medication-related osteonecrosis of the jaw related to epacadostat and pembrolizumab	[70]
CR	2020	Jan-Dirk Raguse; Andrej Trampuz; Marcelo Sanchez Boehm; Susanne Nahles; Benedicta Beck-Broichsitter; Max Heiland; Norbert Neckel	Replacing one evil with another: Is the fibula really a dispensable spare part available for transfer in patients with medication-related osteonecrosis of the jaws?	[71]
CR	2010	Jang-Jaer Lee; Shih-Jung Cheng; Jiiang-Huei Jeng; Chun-Pin Chiang; Hon-Ping Lau; Sang-Heng Kok	Successful Treatment of Advanced Bisphosphonate-related Osteonecrosis of the Mandible With Adjunctive Teriparatide Therapy	[72]
CS	2023	Joel B. Epstein; Praveen R. Arany; Susan E. Yost; YuanYuan	Medication-Related Osteonecrosis of the Jaw: Successful Medical Management of Complex Maxillary Alveolus with Sinus Involvement	[73]
CR	2019	Jun-Young Kim; Jin Hoo Park; Hwi-Dong Jung; Young-Soo Jung	Treatment of Medication-Related Osteonecrosis of the Jaw Around the Dental Implant With a Once-Weekly Teriparatide: A Case Report and Literature Review	[74]
CS	2022	Karen Maciel Reyes Castillo; Miguel Ángel Ocampo Benítez; Omar Peña Curiel	Medication-related osteonecrosis of the Jaw in Cancer Patients: Case Series and Review of the Current Literature	[75]
CR	2020	Keiichi Ohta; Hitoshi Yoshimura	Medication-Related Osteonecrosis of the Jaw	[76]
CR	2020	Kazuki Kiho; Shinichiro Sumitomo; Masashi Tanaka; Tomoya Hasegawa; Chinami Sakai; Yoshiaki Takitani; Yoshida; Satoshi Kawano	Pulpal Disease Arising from Medication-related Osteonecrosis of the Jaw: A Case Report	[77]
CR	2021	Adarsh Kudva; Jonathan Koshy; Joanna Grace Jacob	Oral mucosal pseudotumor–Novelty complication in patient undergoing bevacizumab therapy	[78]
CR	2014	Kyo-Jin Ahn; Young-Kyun Kim; Pil-Young Yun	Reconstruction of Defect after Treatment of Bisphosphonate-related Osteonecrois of the Jaw with Staged Iliac Bone Graft	[79]
CS	2017	Johannes Laimer; Otto Steinmass; Hechenberger; Michael Rasse; Rajmond Pikula; Emanuel Bruckmoser	Intraoral Vacuum-Assisted Closure Therapy—A Pilot Study in Medication-Related Osteonecrosis of the Jaw	[80]
CR	2016	Alexandra Johanna Leven; Antony J Preston	Conservative Prosthetic Rehabilitation of Medication Related Osteonecrosis of the Jaw (MRONJ)	[81]
CS	2015	Linas Zaleckas; Mindaugas Stacevičius; Dovilė Proškutė; Jurgita Povilaitytė	Medication-related osteonecrosis of the jaws. The first reported cases in the Baltic States and a literature review	[82]
CR	2021	Lokendra Gupta; Kanchan Dholam; Yogesh Janghel; Sandeep V. Gurav	Osteonecrosis of the jaw associated with imatinib therapy in myeloproliferative neoplasm: a rare case report	[83]
CR	2020	Louise Dunphy; Giovanni Salzano; Barbara Gerber; Jennifer Graystone	Medication-related osteonecrosis (MRONJ) of the mandible and maxilla	[84]
CR	2021	Luis Monteiro; Catarina Vasconcelos; José-Júlio Pacheco; Filomena Salazar	Photobiomodulation laser therapy in a Lenvatinib-related osteonecrosis of the jaw: A case report	[85]
CS	2017	M. Badr; E. Kyriakidou; A. Atkins; S. Harrison	Aggressive denosumab-related jaw necrosis—a case series	[86]
CR	2023	Maan Ahmad Rafik Asfour; Abeer Ahmad Aljoujou; Maher Sadik Saifo; Haya A. L. Jabban	The use of advanced-platelet rich fibrin (A-PRF) in the management of medication-related osteonecrosis of the jaw (MRONJ): A case report	[87]
CR	2015	Mayuka Maeda; Takeshi Matsunobu; Takaomi Kurioka; Akihiro Kurita; Akihiro Shiotani	A case of nasal septal abscess caused by medication related osteonecrosis in breast cancer patient	[88]
CR	2021	Makiko Okubo-Sato; Kenji Yamagata; Satoshi Fukuzawa; Kazuhiro Terada; Fumihiko Uchida; Naomi Ishibashi-Kanno; Hiroki Bukawa	Medication-Related Osteonecrosis of the Jaw Spontaneously Occurred in a Patient with Chronic Myelogenous Leukemia Only by Imatinib: A Report of a Rare Case	[89]
CR	2021	Marcelo Vieira da Costa Almeida; Antonio C. Moura; Nascimento Távora Cavalcanti; Kaline Romeiro	Photodynamic Therapy as an adjunct in the Treatment of Medication-Related Osteonecrosis of the Jaw: A Case Report	[90]
CR	2017	Mario Migliario; Giovanni Mergoni; Paolo Vescovi; Iolanda De Martino; Manuela Alessio; Luca Benzi; Filippo Renò; Vittorio Fusco	Osteonecrosis of the Jaw (ONJ) in Osteoporosis Patients: Report of Delayed Diagnosis of a Multisite Case and Commentary about Risks Coming from a Restricted ONJ Definition	[91]
CR	2022	Masaki Tatsumura; Takeshi Saito; Hiroyuki Ito; Kousei Miura; Masashi Yamazaki	The Decalcification of Cervicothoracic Spinal Metastasis of Breast Cancer Due to Discontinuation of Denosumab: A Case Report	[92]
CR	2019	Mathangi Kumar; Ravindranath Vineetha; Adarsh Kudva	Medication related osteonecrosis of jaw in a leukemia patient undergoing systemic arsenic trioxide therapy: A rare case report	[17]
CS	2015	Matthias Troeltzsch; Florian Probst; Markus Troeltzsch; Michael Ehrenfeld; Sven Otto	Conservative management of medication-related osteonecrosis of the maxilla with an obturator prosthesis	[93]
CR	2023	Mitsunobu Otsuru; Masahiro Umeda; Sakiko Soutome; Saki Hayashida; Kota Morishita; Souichi Yanamoto	Medication-related osteonecrosis of the upper jaw involving the zygomatic bone: A case report	[94]
CR	2015	H. Miyashita; H. Shiba; H. Kawana; T. Nakahara	Clinical utility of three-dimensional SPECT/CT imaging as a guide for the resection of medication-related osteonecrosis of the jaw	[95]
CR	2016	Mürüde Yazan; Fethi Atil; Ismail Doruk Kocyigit; Umut Tekin; Hakan Hifzi Tuz; Melda Misirlioglu	Spontaneous Healing of Mandibular Noncontinuous Defect Caused by Medication-Related Osteonecrosis of the Jaw	[96]
CR	2022	Muhanad MS Mohamed; Wiranthi MA Gunasekera; David Glew; Christopher Bell; Ashok K Bhalla	Teriparatide therapy for medication-related osteonecrosis of the jaw: case report and literature review	[97]
CR	2018	Noritaka Ohga; Jun Sato; Takuya Asaka; Masahiro Morimoto; Yutaka Yamazaki; Yoshimasa Kitagawa	Successful conservative treatment of jaw osteonecrosis caused by denosumab in patients with multiple bone metastasis	[98]
CR	2019	Onur Şahin; Onur Odabas¸i; Ceren Ekmekciog˘lu	Ultrasonic Piezoelectric Bone Surgery Combined With Leukocyte and Platelet-Rich Fibrin and Pedicled Buccal Fat Pad Flap in Denosumab-Related Osteonecrosis of the Jaw	[99]
CR	2018	Pier Paolo Poli; Francisley; Ávila Souza; Carlo Maiorana	Adjunctive use of antimicrobial photodynamic therapy in the treatment of medication-related osteonecrosis of the jaws: A case report	[100]
CS	2023	Robin Kasper; Mario Scheurer; Sebastian Pietzka; Andreas Sakkas; Alexander Schramm; Frank Wilde; Marcel Ebeling	MRONJ of the Mandible—From Decortication to a Complex Jaw Reconstruction Using a CAD/CAM-Guided Bilateral Scapula Flap	[101]
CR	2020	Sahand Samieirad; Ali Labafchi; Khashyar Famili; Haleh Hashemzadeh	Medication-Related Osteonecrosis of the Jaw (MRONJ) due to Simvastatin: An Unusual Case Report	[16]
CR	2021	Keisuke Seki; Shunsuke Namaki; Atsushi Kamimoto; Yoshiyuki Hagiwara	Medication-Related Osteonecrosis of the Jaw Subsequent to Peri-Implantitis: A Case Report and Literature Review	[102]
CR	2020	Shinpei Matsuda; Hisato Yoshida; Minako Shimada; Hitoshi Yoshimura	Spontaneous regeneration of the mandible following hemimandibulectomy for medication related osteonecrosis of the jaw	[103]
CS	2016	Gayathri Subramanian; Evelyne Kalyoussef; Meredith Blitz-Goldstein; Jessenia Guerrero; Nasrin Ghesani; Samuel Y.P. Quek	Identifying MRONJ-affected bone with digital fusion of functional imaging (FI) and cone-beam CT (CBCT)–case reports and hypothesis	[104]
CR	2018	Taiki Suzuki; Ryo Sekiya; Yuji Hamada; Miho Takahashi; Kazunari Karakida; Haruo Sakamoto	Fatal Bleeding in Conjunction with Mandibular Medication-related Osteonecrosis of the Jaw (MRONJ)	[105]
CR	2017	Ayano Taniguchi; Keita Fukazawa; Toyoshi Hosokawa	Selective Percutaneous Controlled Radiofrequency Thermocoagulation of the Gasserian Ganglion to Control Facial Pain Due to Medication-Related Osteonecrosis of the Jaw	[106]
CS	2016	Tarek Metwally; Andrea Burke; Jeffrey Y Tsai; Michael T Collins; Alison M Boyce	Fibrous dysplasia and medication-related osteonecrosis of the jaw	[107]
CS	2024	Tito Lúcio Fernandes; Bruno Viezzer Fernandes; Gilson Cesar Nobre Franco	Treatment of Medication-Related Osteonecrosis of the Jaws without Segmental Resections: A Case Series	[108]
CR	2021	Tomasz Matys	Medication-related Osteonecrosis of the Jaw	[109]
CR	2021	Yoshinari Myoken; Yoshinori Fujita; Ryota Imanaka; Shigeaki Toratani	Bosutinib-induced osteonecrosis of the jaw in a patient with chronic myeloid leukemia: a case report	[110]
CR	2020	Yoshinari Myoken; Yoshinori Fujita; Kazuma Kawamoto; Shigeaki Toratani	Osteonecrosis of the jaw in a metastatic lung cancer patient with bone metastases undergoing pembrolizumab + denosumab combination therapy: Case report and literature review	[111]
CR	2016	P. Zarringhalam; E. Brizman; K. Shakib	Medication-related osteonecrosis of the jaw associated with aflibercept	[112]

## Appendix B

**Table A2:** Summary of the type of treatment instituted and the existence, or not, of a cure for MRONJ in the included articles.

Case	Antibiotics	Mouthwash	Surgery	Others	Cure
1	Yes	Yes	No	No	Yes
2	Yes	No	Yes	No	Yes
3	Yes	Yes	No	No	Yes
4	Yes	Yes	Yes	Yes	Yes
5	Yes	Yes	Yes	Yes	No
6	Yes	Yes	Yes	Yes	No
7	No	No	Yes	No	Yes
8	Yes	No	Yes	No	No
9	Yes	Yes	Yes	No	Yes
10	Yes	Yes	Yes	No	Yes
11	Yes	No	Yes	No	No
12	Yes	Yes	Yes	No	Yes
13	Yes	Yes	No	No	No
14	Yes	Yes	Yes	No	Yes
15	Yes	Yes	Yes	No	Yes
16	Yes	Yes	No	Yes	No
17	No	No	Yes	No	Yes
18	No	No	Yes	No	No
19	Yes	Yes	No	Yes	Yes
20	No	Yes	Yes	No	No
21	Yes	Yes	No	No	No
22	Yes	Yes	No	Yes	No
23	Yes	No	Yes	Yes	No
24	Yes	Yes	Yes	Yes	No
25	Yes	No	No	No	No
26	Yes	Yes	Yes	Yes	Yes
27	No	No	No	Yes	Yes
28	Yes	Yes	Yes	Yes	Yes
29	Yes	No	Yes	No	No
30	Yes	No	Yes	No	No
31	Yes	Yes	Yes	No	Yes
32	No	No	No	Yes	Yes
33	Yes	Yes	Yes	Yes	No
34	Yes	No	Yes	Yes	Yes
35	No	No	No	Yes	Yes
36	Yes	Yes	No	No	Yes
37	Yes	Yes	Yes	No	No
38	Yes	Yes	Yes	Yes	Yes
39	Yes	Yes	No	Yes	Yes
40	Yes	Yes	Yes	No	Yes
41	Yes	Yes	Yes	No	Yes
42	No	No	Yes	No	Yes
43	No	No	Yes	Yes	Yes
44	Yes	Yes	Yes	Yes	Yes
45	Yes	Yes	Yes	Yes	No
46	Yes	Yes	Yes	Yes	Yes
47	Yes	No	Yes	No	Yes
48	Yes	Yes	Yes	No	No
49	Yes	No	No	Yes	Yes
50	Yes	Yes	Yes	No	Yes
51	Yes	Yes	No	No	No
52	Yes	Yes	Yes	Yes	Yes
53	Yes	Yes	Yes	Yes	Yes
54	Yes	No	Yes	Yes	Yes
55	Yes	Yes	Yes	No	No
56	Yes	Yes	No	No	No
57	Yes	Yes	Yes	Yes	No
58	Yes	Yes	No	No	No
59	Yes	Yes	Yes	Yes	No
60	No	No	Yes	No	Yes
61	No	Yes	Yes	Yes	Yes
62	No	No	Yes	No	No
63	Yes	Yes	Yes	No	No
64	Yes	Yes	Yes	No	Yes
65	Yes	Yes	Yes	No	Yes
66	Yes	Yes	Yes	Yes	Yes
67	Yes	Yes	Yes	Yes	Yes
68	Yes	Yes	No	No	No
69	Yes	Yes	No	Yes	Yes
70	Yes	Yes	Yes	No	Yes
71	No	No	Yes	Yes	Yes
72	Yes	No	Yes	Yes	No
73	No	Yes	Yes	No	Yes
74	Yes	Yes	Yes	No	No
75	No	Yes	No	No	No
76	Yes	Yes	Yes	Yes	No
77	Yes	No	Yes	No	Yes
78	Yes	Yes	Yes	No	Yes
79	Yes	Yes	Yes	Yes	Yes
80	Yes	Yes	Yes	No	Yes
81	Yes	Yes	Yes	Yes	No
82	Yes	Yes	Yes	No	No
83	No	No	Yes	Yes	Yes
84	Yes	Yes	No	No	Yes
85	Yes	Yes	Yes	Yes	No
86	Yes	No	Yes	No	No
87	No	No	No	Yes	Yes
88	Yes	No	Yes	No	No
89	No	No	Yes	No	Yes
90	Yes	Yes	Yes	Yes	Yes
91	Yes	Yes	Yes	No	Yes
92	Yes	Yes	Yes	Yes	Yes
93	Yes	Yes	No	No	No
94	Yes	No	Yes	Yes	Yes
95	Yes	Yes	Yes	No	No
96	Yes	Yes	Yes	Yes	No
97	Yes	Yes	Yes	Yes	Yes
98	Yes	Yes	No	No	No
99	Yes	Yes	Yes	No	No
100	Yes	No	No	No	No
101	Yes	Yes	No	No	Yes
102	Yes	Yes	Yes	No	No
103	Yes	No	No	Yes	Yes
104	Yes	No	Yes	No	No
105	Yes	No	Yes	Yes	Yes
106	Yes	Yes	Yes	No	Yes
107	Yes	No	Yes	Yes	No
108	Yes	No	Yes	No	No
109	Yes	Yes	Yes	No	No
110	Yes	Yes	Yes	No	Yes
111	Yes	Yes	No	No	Yes
112	No	Yes	Yes	No	No
113	Yes	Yes	Yes	No	Yes
114	Yes	No	Yes	No	No
115	Yes	No	Yes	No	No
116	Yes	No	Yes	No	No
117	Yes	No	Yes	No	No
118	No	No	Yes	No	Yes
119	Yes	No	Yes	No	No
120	No	Yes	No	No	No
121	Yes	Yes	No	Yes	Yes
122	Yes	Yes	No	No	Yes
123	Yes	Yes	No	No	Yes
124	Yes	Yes	No	Yes	Yes
125	Yes	No	Yes	Yes	Yes
126	Yes	Yes	Yes	Yes	Yes
127	Yes	Yes	Yes	Yes	Yes
128	No	No	No	Yes	No
129	No	No	Yes	No	Yes
130	Yes	No	Yes	Yes	Yes
131	Yes	No	Yes	Yes	Yes
132	Yes	No	Yes	Yes	Yes
133	Yes	No	Yes	Yes	Yes
134	Yes	No	Yes	Yes	Yes
135	Yes	No	Yes	Yes	Yes
136	Yes	No	Yes	Yes	Yes
137	Yes	No	Yes	Yes	Yes
138	Yes	No	Yes	Yes	Yes
139	Yes	Yes	Yes	Yes	Yes
140	Yes	No	Yes	No	No
141	Yes	No	Yes	No	Yes
142	Yes	Yes	Yes	Yes	Yes
143	Yes	Yes	Yes	No	No
144	Yes	No	Yes	No	No
145	Yes	Yes	Yes	Yes	Yes
146	Yes	Yes	Yes	Yes	Yes
147	Yes	Yes	Yes	Yes	Yes
148	Yes	Yes	Yes	Yes	Yes
149	Yes	Yes	Yes	Yes	No
150	Yes	Yes	Yes	Yes	Yes
151	Yes	No	Yes	Yes	Yes
